# Polymorphic variant at the *IL2* region is associated with type 1 diabetes and may affect serum levels of interleukin-2

**DOI:** 10.1007/s11033-013-2815-9

**Published:** 2013-10-24

**Authors:** Marta Fichna, Magdalena Żurawek, Piotr Fichna, Iwona Ziółkowska-Suchanek, Danuta Januszkiewicz, Jerzy Nowak

**Affiliations:** 1 Institute of Human Genetics, Polish Academy of Sciences, 32 Strzeszynska, 60-479 Poznan, Poland; 2 Department of Endocrinology and Metabolism, Poznan University of Medical Sciences, 49 Przybyszewskiego, 60-355 Poznan, Poland; 3 Department of Pediatric Diabetes and Obesity, Poznan University of Medical Sciences, 27/33 Szpitalna, 60-572 Poznan, Poland; 4 Department of Pediatric Oncology, Hematology and Transplantology Poznan University of Medical Sciences, 27/33 Szpitalna, 60-572 Poznan, Poland

**Keywords:** *IL2* gene, Polymorphism, Interleukin-2, Type 1 diabetes

## Abstract

Polymorphic variants at the interleukin-2 (*IL2*) locus affect the risk of several autoimmune disorders. Our aim was to evaluate the association of the four *IL2* polymorphisms (rs6822844, rs6534349, rs2069762 and rs3136534) with type 1 diabetes (T1D) in the Polish population, and to correlate them with the serum interleukin-2 levels. 543 unrelated T1D patients and 706 healthy control subjects were enrolled. The minor T allele at rs6822844 was significantly less frequent in T1D compared to controls (*p* = 0.002; OR 0.71; 95 % CI 0.571–0.880). Likewise, the frequency of the TT genotype was decreased among the affected individuals (*p* = 0.007). In healthy subjects, stratification according to the rs6822844 genotype revealed significant differences in circulating interleukin-2 (*p* = 0.037) with the highest levels in TT protective genotypes. Three other *IL2* polymorphisms did not display significant differences in allele and genotype distribution. In conclusion, the rs6822844 variant is associated with T1D and may play a functional role, or reflect the influence of another causative genetic variant in linkage disequilibrium.

## Introduction

Type 1 diabetes (T1D) results from selective destruction of the insulin-secreting pancreatic beta cells by the autoreactive T lymphocytes. The disease onset is probably triggered by unknown environmental factors (diet, infection) superimposed on specific genetic background in susceptible individuals. To date several genetic loci have been identified which may affect the immune function and thereby contribute to T1D development. The strongest effect was demonstrated for the HLA region (chromosome 6p21), but other genes involved in the immune response also play a role [1]. The *IL2* gene, which encodes interleukin-2, a cytokine produced predominately by the activated CD4+ and CD8+ T cells in response to T cell receptor (TCR) stimulation, was one of the strong functional candidates. Plausible association of the *IL2* gene with diabetes was first suggested in non-obese diabetic (NOD) mouse model, based on congenic mapping of the Idd3 susceptibility locus [2]. Functional data revealed that *IL2* variants in NOD mice might affect the interleukin-2 production by antigen-specific T cells and predispose to organ-specific autoimmunity [3]. These analyses were followed by the first genome-wide association scan in human T1D, which showed a moderate evidence of the association with the *IL2* region (4q27) [4]. Rs6534347, an intronic variant of the nearby *KIAA1109* gene, was identified by imputation and rs17388568, localized to an intron in the adjacent *TENR* (*ADAD1*) gene, revealed the strongest signal based on multilocus analysis [4]. Further sequencing and genotyping of the *IL2* region in T1D cohorts revealed the associations with rs3136534, rs6836189 and replicated an association with rs17388568, however, due to extensive linkage disequilibrium (LD) across this locus, the genuine causative variant could not be determined [5].

Early on, interleukin-2 was mainly regarded as a core cytokine in protective cell-mediated immunity, responsible for the clonal expansion of the effector T cells. Nowadays its major and non-redundant action is attributed to the development and homeostasis of the regulatory CD4+CD25+Foxp3+ T (Treg) cells, which ensure the elimination of the self-reactive T lymphocytes [6]. Interleukin-2-deficient mice develop a lethal lymphoproliferative syndrome with massive inflammatory lesions and detectable autoantibodies [7]. There is increasing body of in vitro evidence to support the crucial role of the IL2 signaling pathway in Treg cell function and maintenance of self-tolerance [8]. Therefore, polymorphic variants at the 4q27 locus, which might alter the *IL2* expression, could consequently affect the work of the immune system. Indeed, single nucleotide polymorphisms (SNPs) in this region were found to be associated with systemic and organ-specific autoimmune disorders, such as rheumatoid and juvenile idiopathic arthritis, multiple sclerosis, psoriasis, celiac disease, inflammatory bowel disease [9–15]. Allergic conditions—IgE-mediated allergy, asthma and atopic dermatitis—also displayed an association with the *IL2* gene variants [16]. Moreover, *IL2* SNPs were linked with other immune phenomena, such as measles vaccine-induced immunity and the risk of graft-versus-host disease in patients undergoing hematopoietic stem cell transplantation [17, 18]. However, despite the unequivocal association with other autoimmune disorders and promising GWAS results, 4q27 locus has been rarely examined in the replication studies in different T1D populations to date [9, 10, 19].

The aim of this study was to investigate the association of selected polymorphic variants of the *IL2* gene (rs6822844 G/T, rs6534349 A/G, rs2069762 G/T, and rs3136534 A/C) with T1D in the Polish population. We also made an attempt to correlate the genotyping results with serum levels of the interleukin-2, which may represent an intermediate phenotype, directly affected by the genetic factors.

## Materials and methods

### Selection of investigated *IL2* SNPs

The choice of investigated polymorphisms was based upon previous reports showing their involvement in T1D and other autoimmune disorders. Rs6822844, located between the *IL2* and the neighboring *IL21* gene, was found to be associated with celiac disease, inflammatory bowel disease, psoriasis, juvenile idiopathic and rheumatoid arthritis [9–13, 15]. Rs2060762, located upstream to the *IL2* promoter (at −330 site), was previously shown to associate with autoimmune (multiple sclerosis, MS) and allergic conditions [14, 16]. The choice of rs3136534 was supported by the data from the first genome-wide association study in T1D [5]. Finally, rs6534349 was selected as a tagger (*r* ^2^ > 0.9) for two other *IL2* SNPs, rs2069777 and rs2069779, located in intron 2 and 3, respectively.

### Genotyping

The studied cohort consisted of 543 unrelated T1D patients (284 females, 259 males) and 706 healthy control subjects (378 females, 328 males), from the local Polish population of Caucasian origin. Patients were recruited at the Department of Pediatric Diabetes and Obesity. Their mean age (±SD) at disease onset was 8.6 ± 4.5 years. The diagnosis of T1D was based upon World Health Organization criteria and all patients required lifetime insulin substitution. Control samples for the genotyping were obtained from healthy blood donors with negative history of autoimmunity and no signs of autoimmune disorders, recruited at the Regional Blood Transfusion Centre. Their mean age was 38.1 ± 10.2 years.

Genomic DNA was extracted from the peripheral blood using Gentra Puregene Blood Kit (Qiagen). Genotyping was performed using the polymerase chain reaction followed by restriction fragment length polymorphism analysis (PCR–RFLP) for SNPs rs6534349, rs2069762 and rs3136534. All fragments were amplified in 30-μl PCR reactions using primers designed with the help of Primer3 online tool, and manufactured by Oligo (Warsaw, Poland) [20]. Primer sequences, PCR conditions, restriction enzymes and fragments are presented in Table 1. Genotyping of the rs6822844 was performed by allelic discrimination analysis using the 7900HT Real-Time PCR System and Taqman assay (C_28983601_10), under the conditions recommended by the manufacturer (Applied Biosystems, Foster City, CA). All genotypes were confirmed in 5 % of samples by direct DNA sequencing with a BigDye Terminator Cycle Sequencing Ready Reaction Kit (ABI Prism 3730 Genetic Analyzer, Foster City, CA). The samples of confirmed genotypes were run as controls in all reactions and 10–12 % of samples were genotyped twice.

**Table 1:** Primer sequences, PCR conditions and restriction enzymes used for genotyping of the *IL2* SNPs: rs6534349, rs2069762, rs3136534

SNP	Primers	Amplico*n* size (bp)	Annealing temperature	Restriction enzyme	Restriction fragments size (bp)
rs6534349 A/G	F: 5′CCTACTGTTTCTAGTTACTG	437	49 °C	BstNI (MvaI)	Allele A: 84 + 353
	R: 5′AGATTGGGACTATAAGACTG				Allele G: 84 + 153 + 200
rs2069762 T/G	F:5′ATTCACATGTTCAGTGTAGTTCTA	229	50 °C	BfaI (FspBI)	Allele T: 229
	R: 5′TCCTCTTCTGATGACTCTTTG				Allele G: 22 + 207
rs3136534 A/C	F: 5′GCCAAGGCTCTAGGTGAACA	359	57 °C	PsuI (BstYI)	Allele A: 359
	R: 5′CAAGCTCTGCCTACCAGGTT				Allele C: 87 + 272

The forward primer of the rs2069762 was modified (T→C) in order to include a BfaI restriction site

### Serum interleukin-2 levels

Serum levels of interleukin-2 were determined in a subgroup of 93 consecutive patients with newly diagnosed T1D (mean age 8.9 ± 4.7 years) and 84 age-matched healthy individuals (mean age 8.4 ± 5.3). Samples were collected on the 20–24th day after T1D diagnosis, when the affected subjects reached metabolic homeostasis on insulin substitution, and no signs of inflammation were detectable (confirmed by a negative serum C-reactive protein). Control samples for serum interleukin comparisons were collected from healthy children undergoing routine medical checkup before prophylactic vaccinations. Sera were obtained after overnight fast and stored at −70 °C until analyzed. The cytokine levels were evaluated by means of an ELISA assay (Quantikine^®^ Human IL-2 Immunoassay, R&D Systems, Inc. Minneapolis, MN). The detection limit for interleukin-2 is less than 7 pg/ml and intra- and inter-assay precision CV 2.0–4.3 and 3.7–5.0 %, respectively.

The study was approved by the Ethics Committee at Poznan University of Medical Sciences, and informed consent was obtained from all participants or from their parents in case of minors.

### Statistical analysis

Hardy–Weinberg equilibrium was tested for each studied SNP using online calculator available at the Helmholtz Center Munich website (http://ihg.gsf.de/cgi-bin/hw/hwa1.pl). Patients and the control group were compared by ${{\upchi}}^{2}$ tests using 2 × 2 and 3 × 2 contingency tables of genotype and allele counts, respectively. The differences were considered significant with a *p* value <0.05. A Bonferroni correction for multiple test (four independent SNPs) would require a significance threshold of *p* < 0.0125. Logistic regression was used to determine the allelic odds ratios (ORs) and their 95 % confidence intervals (95 % CI) in case–control comparisons. The calculations were performed using SPSS 18.0 software (SPSS Inc., Chicago, IL). Linkage disequilibrium (LD) measures (*r* ^2^) between investigated *IL2* markers were assessed with Haploview v.4.1 [21]. The power of the study to detect an association was calculated with the PS Power and Sample Size calculator v.2.1.30 (http://www.mc.vanderbilt.edu/prevmed/ps). Assuming an OR of 0.64, the power of the present study to detect an effect in the T1D cohort was 98 % for rs6822844 [9]. The power to reproduce an OR of 1.54 previously reported for rs2069762 in MS was 92 % [22], and to replicate an OR of 1.11 found for rs3136534 in T1D–only 31 % [5, 9].

Serum levels of interleukin-2 are presented as mean ± standard deviation (±SD) in case of a normal data distribution or as median with their range when the distribution was not normal. Kolmogorov–Smirnov test was used to check for the data normality. Normally distributed interleukin-2 levels in patients and controls were compared using unpaired Student’s *t* test, while genotype-stratified subgroups displayed non-normal data distribution and hence were analysed by Kruskal–Wallis test. Two-tailed *p* values < 0.05 were considered statistically significant.

## Results

### Genotyping

No SNP displayed a significant departure from Hardy–Weinberg equilibrium in the patient and control cohorts (*p* ≥ 0.074).

The minor T allele at the rs6822844 locus was found in 14.2 % (154 of 1,085) T1D alleles compared to its 18.9 % prevalence (267 of 1,412 alleles) in healthy controls (*p* = 0.002), yielding a protective OR of 0.71 (95 % CI 0.571–0.880). In line, the frequency of the TT genotype was significantly lower among affected subjects *vs*. controls (*p* = 0.007) (Table 2). Both associations survived after correction for multiple testing (4 tests, corrected *p*-values 0.008 and 0.028, respectively). The other three SNPs: rs6534349, rs2069762, and rs3136534, did not present significant associations in these investigated groups (complete allele and genotype data displayed in Table 2). Linkage disequilibrium measurements revealed very low pairwise *r* ^2^ values between rs6822844 and other *IL2* SNPs, ranging from 0.01 (rs6822844–rs6534349) to 0.09 (rs6822844–rs3136534). Similar LD results were derived from the HapMap CEU genotype data. Therefore, the haplotype analysis was not performed, as the study of the inferred haplotypes would not add any further information.

**Table 2:** Distribution of the *IL2* genotypes and alleles in Polish patients with type 1 diabetes (T1D) and controls

*IL2* SNP	Genotype	Allele	T1D patients	Controls	Uncorrected *p* value
			*n* = 543 (%)	*n* = 706 (%)	
rs6822844 G/T	GG		405 (74.6)	468 (66.3)	**0.007**
	GT		122 (22.5)	209 (29.6)	
	TT		16 (2.9)	20 (4.1)	
		G	932 (85.8)	1,145 (81.1)	**0.002**
		T	154 (14.2)	267 (18.9)	
rs6534349 A/G	AA		440 (81.0)	578 (81.9)	0.562
	AG		97 (17.9)	124 (17.5)	
	GG		6 (1.1)	4 (0.6)	
		A	977 (90.0)	1,280 (90.7)	0.564
		G	109 (10.0)	132 (9.3)	
rs2069762 G/T	TT		273 (50.2)	339 (48.0)	0.604
	GT		217 (40.0)	302 (42.8)	
	GG		53 (9.8)	64 (9.2)	
		T	763 (70.3)	980 (69.4)	0.646
		G	323 (29.7)	432 (30.6)	
rs3136534 A/C	AA		225 (41.4)	281 (39.8)	0.840
	AC		259 (47.7)	345 (48.9)	
	CC		59 (10.9)	80 (11.3)	
		A	709 (65.3)	907 (64.2)	0.586
		C	377 (34.7)	505 (35.8)	

*P* values in bold indicate statistically significant results

### Serum interleukin-2 levels

Serum interleukin-2 levels in T1D patients were lower compared to controls however, the difference did not reach statistical significance (8.80 ± 4.95 vs. 10.66 ± 5.42 pg/ml, *p* = 0.066). Stratification of the control samples according to the rs6822844 genotype revealed significant difference in the interleukin-2 concentration: median 7.94 (range 0.07–17.52) pg/ml for GG, 9.28 (5.25–18.66) pg/ml for GT and 13.80 (6.82–20.83) pg/ml for TT (*p* = 0.037). On the contrary, circulating interleukin-2 levels in T1D patients were similar in carriers of different rs6822844 genotypes (*p* = 0.358) (Table 3).

**Table 3:** Serum levels of interleukin-2 in patients with type 1 diabetes (T1D) and in healthy control subjects stratified according to the rs6822844 genotype

Interleukin-2 (pg/ml)	rs6822844 genotype			*p*-value
	GG	GT	TT	
T1D patients	10.07 (0.07–20.83)	11.60 (4.48–17.90)	12.19 (5.60–19.67)	0.358
Controls	7.94 (0.07–17.52)	9.28 (5.25–18.66)	13.80 (6.82–20.83)	**0.037**

Interleukin values represent medians (range), and *p* values refer to nonparametric Kruskal–Wallis test comparing genotype-stratified subgroups

*P* values in bold indicate statistically significant results

## Discussion

The current study indicates an association between rs6822844 polymorphism and T1D in the Polish population. In our analysis, the minor T allele appeared protective, with only 2.9 % of T1D patients carrying the TT genotype compared to 4.1 % of healthy controls. These results confirm formerly published data from the Dutch and Columbian T1D cohorts [9, 10]. On the contrary, in a study of genetic variants shared between T1D and celiac disease, rs6822844 did not reach the threshold of statistical significance in a large British T1D cohort [23]. Likewise, a recent analysis among Spanish T1D subjects revealed only a trend for significance at this SNP [19]. These discrepancies may arise from the population differences, or limited sample size, which might be insufficient to detect a modest effect. However, the direction of this association, with a protective T allele and a major G allele being a risk factor, is common to other autoimmune phenotypes, comprising celiac disease, ulcerative colitis, juvenile idiopathic and rheumatoid arthritis [9, 11–13]. Previous and current findings provide ample evidence for the 4q27 region as a general autoimmunity locus. This 480 kb region harbors four genes: *KIAA1109*–*TENR*–*IL2*–*IL21*. *KIAA1109* is highly expressed in ovaries and brain, *TENR* expression is testis-specific, whereas *IL2* and *IL21* encode interleukins, which are both functional candidates in autoimmune diabetes. Interleukin-21 is another T cell-derived cytokine, which enhances the proliferation and effector role of CD8+ T lymphocytes, and is required for generating an inflammatory Th17 response [24]. Priming with interleukin-21 enables autoreactive CD8+ T cells to respond to weak antigens and may induce the diabetes onset in susceptible NOD mice [25].

Rs6822844 is a noncoding SNP, located downstream to the *IL2* and approximately 24 kb 5′ of the *IL21* gene. Based on sequence homology, it was suggested that the rs6822844-carrying fragment might encode a micro-RNA precursor [10]. A substitution of the conserved G allele by the minor T variant could hamper miRNA synthesis, and consequently influence the expression of other genes. In fact, the true role of this SNP remains to be characterized. One may not exclude that a genuine causal variant, which affects the immune function and contributes to autoimmunity, is located elsewhere within a cluster of several SNPs in the 4q27 region. Rs6822844 was reported as a perfect proxy for the five-SNP haplotype (comprising rs4505484, rs11732095, rs6822844, rs4492018 and rs1398553) that is most associated with autoimmune disease [9]. In contrast, LD between rs6822844 and another T1D-associated SNP, rs17388568, is low (*r* ^2^ = 0.09) hence both variants might exert an independent effect. However, localization of rs17388568 in intron 8 of the *TENR* gene makes its direct influence on immune system rather unlikely.

We did not find any association with three other studied SNPs. Our analysis was underpowered to reliably detect an effect of rs3136534. In contrast, the power of this study was adequate to investigate rs2069762, a polymorphic variant consistently associated with MS [22]. Although T1D and MS are both autoimmune conditions, disturbances of different immune pathways might prevail in their development. For instance, distinct causal SNPs in the 4q27 locus could affect the expression of *IL2* and *IL21*. On the other hand, if susceptibility variants located within the same locus do not overlap, this may reflect the causative role of yet another SNP, which remains in LD. To elucidate this matter, the region should be thoroughly resequenced and further genotyped in large cohorts in order to identify most plausible SNPs for functional analyses.

Interleukin-2 plays a key role in thymic differentiation and peripheral expansion of CD4 + CD25 + Tregs. Polymorphic variants of the genes encoding interleukin-2 and its receptor subunits might enhance/impair their expression and therefore affect the activity of the IL2 signaling pathway. This in turn could influence the function of the CD4+CD25+ Tregs and eventually, promote the development of autoimmune disorders. In fact, SNPs located within the extended murine *IL2* promoter, which associate with autoimmune disease, were found to alter the gene transcription in CD4+ T cells [26] and diminished interleukin-2 synthesis was reported for a susceptible haplotype in NOD mice [3]. In humans, the presence of an autoimmunity-associated *IL2RA* haplotype correlates with reduced interleukin-2 responsiveness in stimulated CD4+ T cells and decreased ability of Tregs to suppress the proliferation of autologous effector T cells [8].

In our study we limited the analysis to serum levels of interleukin-2 in patients and controls, and thereafter we stratified the results according to *IL2* genotype. Interleukin-2 is a cardinal regulatory cytokine in the immune system, but its pleiotropic role may impair reliable distinction of the genuine effects from secondary changes related with the disease. This is the most likely reason of major inconsistencies between former studies, which revealed various interleukin-2 levels among T1D subjects compared to healthy controls [27–29]. A trend for lower circulating interleukin-2 in T1D was found in our study. This observation remains in line with most previous analyses and is also corroborated by the results of the peripheral blood mononuclear cell ex vivo cultures [27, 28, 30–32]. However, the experiments, which investigated in vitro synthesis of the interleukin-2 in a longitudinal way, indicate that its peak was similar but considerably delayed in T1D subjects compared to controls [33]. These observations support a hypothesis of impaired interleukin-2 secretory pattern among patients with T1D. Of note, the interleukin-2 production by the T1D lymphocytes appears independent of the degree of the metabolic control, and is probably regulated at a pretranslational stage [30, 31]. Moreover, decreased serum interleukin-2 concentrations were also reported in diabetic patients with long-lasting disease, when the active autoimmune reaction has been attenuated [28]. Therefore, at least partial modulation of the interleukin-2 synthesis could be attributed to genetic factors [32]. Former analyses revealed an association between serum cytokine levels and a polymorphic variant upstream to the *IL2* gene promoter (−330 T/G, rs2060762) assessed in MS and in neuroendocrine tumors [14, 34]. Although its position makes rs2060762 a plausible functional candidate, we were not able to confirm this correlation in our cohort (data not shown, *p* > 0.05). Instead, significant difference in circulating interleukin-2 was found among healthy carriers of three rs6822844 genotypes, and the lowest cytokine levels were present in subjects with the T1D risk GG genotype (Table 3). However, the statistical significance of this finding is borderline. Additionally, this relation was no more significant when evaluated among T1D subjects (*p* = 0.358), possibly due to overlying immune phenomena connected with the recent disease onset. Replication studies in larger cohorts, as well as experimental investigation of the *IL2* gene expression at the mRNA level would be mandatory to confirm these findings. Considering the location of the rs6822844 with regard to the *IL2* gene, this SNP seems rather unlikely regulator of the gene expression, however, another unidentified polymorphism in LD may directly affect *IL2* regulation. Gene expression usually undergoes complex, multifactorial influence, but similar genotype-intermediate phenotype relationship was reported for the *IL2RA* gene, which encodes high-affinity alpha subunit of the interleukin-2 receptor (CD25 molecule). Its polymorphic variants are strongly associated with T1D and affect the soluble receptor levels, although discrepancies in the effect of the *IL2RA* genotype on serum measurements were detected in different autoimmune conditions [35].

In conclusion, the present study confirms the association of the rs6822844 with T1D in another Caucasian population. Furthermore, genotype-related differences in serum interleukin-2 levels suggest plausible functional role of this polymorphism. However, other nearby SNPs, which remain in LD with rs6822844, may play the true causative role. Further functional studies are essential to dissect downstream effects of the molecular variants at this locus and the mechanisms of their contribution to increased risk for T1D and other autoimmune pathology.
